# Impact of NPSB fertilizer on yield of orange-fleshed sweet potato (*Ipomoea batatas* (L.) Lam.) varieties in Southern Ethiopia's agro-ecological zones

**DOI:** 10.1016/j.heliyon.2024.e40660

**Published:** 2024-11-23

**Authors:** Amelewerk Gizachew, Sabura Shara, Asfaw Kifle

**Affiliations:** a Raya University, College of Agricultural and Natural Resources, Department of Horticulture, Maichew, P.O. Box 92, Ethiopia; b Arba Minch University, College of Agricultural Sciences, Department of Horticulture, Arba Minch, P.O. Box 21, Ethiopia; c Areka Agricultural Research Center, Plant Science Work Process, Areka, P.O. Box 79, Ethiopia

**Keywords:** Orange-fleshed sweet potato, Variety response, Site-specific fertilizer management, Blended NPSB, Agronomic efficiency, Partial budget analysis, Storage root yield

## Abstract

Root and tuber crops, particularly sweet potatoes, are vital to global food security, yet their potential for enhancing household nutrition and income remains largely untapped. Orange-fleshed sweet potato (OFSP) varieties are rich in pro-vitamin A, crucial for health. Despite their significance in Ethiopia, yields are often low due to inadequate location-specific fertilizer recommendations and limited access to high-yielding varieties. This study aimed to determine the optimal blended NPSB fertilizer rates and high-yielding OFSP varieties in the contrasting environments of Wolaita and Gamo zones, where these crops are staples. We evaluated three OFSP varieties (Alamura, Dilla, and Kabode) and four NPSB rates (0, 79.5, 159, and 238.5 kg ha^−1^ in a 3x4 factorial experiment using a randomized complete block design (RCBD) with three replications during the 2022 cropping season. Data on root yield and yield components were analyzed using the GLM procedure in SAS 9.2. Partial budget analysis and agronomic efficiency were also computed. Results showed that marketable root yield and root dry matter were significantly affected by the interaction of site, fertilizer rates, and OFSP varieties. The highest marketable root yield (49.84 t ha^−1^ and net benefit (342,856.6 ETB ha^−1^) were achieved with the Kabode variety at 238.5 kg NPSB in Arba Minch, while the same variety yielded the highest net benefit from 159 kg NPSB in Areka. The highest agronomic efficiency was recorded at 159 kg NPSB and 79 kg NPSB in Arba Minch (174.71) and Areka (288.67), respectively, indicating a negative relationship between net benefit, agronomic efficiency, and increasing NPSB rates. In conclusion, cultivating the Kabode variety with 159 kg ha^−1^ NPSB in Arba Minch and 79.5 kg ha^−1^ NPSB in Areka is recommended for enhancing sweet potato productivity, thereby improving food security and nutritional benefits for smallholder farmers in the region.

## 1 Introduction

Root and tuber crops are essential for food security of vulnerable communities in the world, especially in sub-Sahara-Africa, Asia and Latin America. Sweet potato (*Ipomoea batatas* (L.) Lam) is globally ranked as the seventh most important food crop, and the third most important root and tuber crop after potato and cassava. In Ethiopia, sweet potato production ranks third among root crops, with a significant presence in regions such as Wolaita, Gamo, Gofa, Sidama, South Omo, Dawro, zones. In addition sweet potato produced in Oromia, Amhara, Afar and Tigray regions. The crop occupies around 62,116.56 ha of land, with an annual production of 1,598,838.49 tons and a productivity of 25.73 tons per hectare during the *Meher* season alone [1]. Orange-fleshed sweet potato (OFSP) varieties are rich vitamin A, which is essential for nutrition and health. Massive efforts have been implemented to integrated OFSP varieties into the food system as one of the accessible and affordable sources of vitamin A in sub-Saharan Africa including Ethiopia, where vitamin A deficiency is a significant public health problem [[2], [3], [4]].

Despite their importance, the productivity of OFSP is variable in the country/southern region and ranges from 17.7 to 35.4 tons per hectare, while implementation of correct fertilizer amendments can achieve yields as high as 63.33 tons per hectare [5]. Generally, the yields of OFSP vary largely with blended NPSB fertilizer rates and OFSP varieties in different environments [6,7]). Variable yields in OFSP in different regions is attributed among other things to mismatch in environments and varieties. This indicates that the yields are low and variable due to lack of knowledge on the production requirements, with soil fertility management and selection of varieties suitable to the local growing conditions being most important.

In Ethiopia, research has focused on the introduction and adaptation of orange-fleshed sweet potato (OFSP), including trials on plant spacing, the use of vine cuttings as planting material, planting positions, optimal harvesting times, and the effects of various chemical fertilizers. These investigations have been carried out in a limited area of the country since 1997 with the aim of enhancing production and productivity [[8], [9], [10]]. However, there has been relatively little emphasis on evaluating OFSP varieties with NPSB fertilizer rates in diverse regions of southern Ethiopia, notably in the Wolaita and Gamo zones. Moreover, the existing literature is sparse regarding the agronomic performance of OFSP varieties with blended NPSB fertilizers in varying environmental conditions. Consequently, there is a lack of location-specific knowledge concerning fertilizer usage and yield potential.

Urea and diammonium phosphate (DAP) were the sole commercial fertilizer sources for nitrogen and phosphorus in Ethiopian agriculture for several decades. About a decade ago [11] revealed the soils, besides N and P, lack other macro- and micro-nutrients such as S, Cu, B, and Zn in Ethiopia in general and the southern region in particular. Subsequently, Ethiopia's Ministry of Agriculture has introduced a new blended NPSB fertilizer, which contains fertilizer grade ratio of 18.9 N + 37.7 P_2_O_5_ + 6.95 S + 0.1 B instead of DAP as the main source of essential nutrients in Ethiopian Agriculture [12]. Economical fertilizer use in agriculture is the one based on soil-test based application. However, regular soil tests would not be affordable by smallholder growers and regional facilities. Thus, the soil fertility status and fertilizer recommendation Atlas of the region indicated only the deficient nutrients at different locations, but recommends the need to determine site-specific fertilizer application rates based on crop-based field trials for this blended NPSB to increase crop productivity in smallholder systems [11,13].

The soils of Wolaita and Gamo zones, known as the main potential areas for sweet potato production in the southern region are deficient in N, P, S, Cu, B, and Zn [14]. However, there is a lack of information on the effects of the NPSB fertilizer on OFSP yield, and yield components in the Wolaita and Gamo zones. To greatly enhance the productivity and quality of storage roots and help resilience of the smallholders systems, it is necessary to fertilize the OFSP with the right amount of nutrients in line with suitable locations. Increasing productivity of such crops will have great roles in increasing food availability and resilience of smallholder farming system to help feed growing population and adapt to the changing climate. Therefore, this study addresses research gaps by examining the impact of blended NPSB rates on sweet potato yield across diverse agro-ecological zones in southern Ethiopia. It aims to fill knowledge voids and offer valuable insights into fertilizer application's effects on sweet potato cultivation, assessing both agronomic efficiency and economic feasibility of these rates on orange-fleshed sweet potato (OFSP) productivity. The findings will enhance understanding of how varying NPSB rates influence sweet potato yield in different agro-ecological settings, ultimately improving agricultural practices and productivity in the region.

## 2 Materials and methods

### 2.1 Study area

**Fig. 1:**
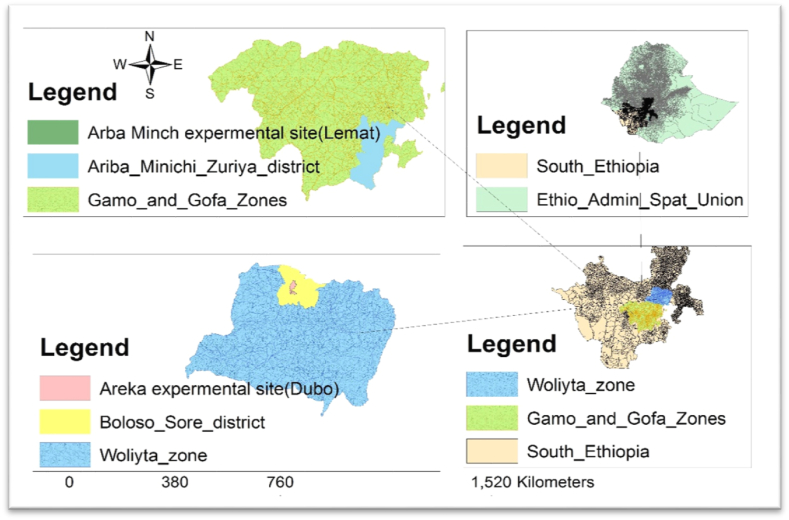
Map of the experimental sites of Wolaita and Gamo zones in Southern Ethiopia.

The field experiment was carried out at Areka agricultural research center in Wolaita zone and the Gamo Development Association Agricultural Farm (GaDAAF) in Gamo zone of southern Ethiopia (Fig. 1). The study was conducted during the 2022 main cropping season (from April 5 to October 30) under rainfed condition. The detail geographic and agro-ecological description of the experimental areas are indicated in Table 1.

**Table 1:** Geographic and agro ecological description of the experimental areas.

Characters	Sites			
	Areka	References	Arba Minch	References
Geographic position	N7° 03′ 53.94″ E37° 41′12.87″	[7]	N6° 03′ 32.98″ E37° 34′ 26.77″	
Altitude (m a.s.l.)	1830	[7]	1230	[8]
Agroecological zones	Midland (Sub-tropical)		Lowland (Tropical)	
Air Temprature (^0^C)	Minimum – 14 Maximum – 26	[9]	Minimum −16 Maximum −37	[10]
Rainfall (mm)	1520	[11]	892	[8]
Soil textural class	Clay loam to silty loam pH 7.2	[7]	Sandy-loam and clay-loam, pH 6.8	[12]

### 2.2 Treatments and experimental design

Treatments consisted of three orange-fleshed sweet potato varieties (Alamura, Kabode, and Dilla) and four blended NPSB fertilizer rates (0, 79.5, 159, and 238.5 kg ha-1) established at both locations, Arba Minch and Areka. Description of orange-fleshed sweet potato (OFSP) varieties used in the experiment is indicated in Table 2. The experimental field was manual cleaned and ploughed three times. It was further worked on through disking and harrowing, utilizing both tractors and hand tools to achieve a depth of 15–30 cm. The plots were leveled, and ridges measuring around 30 cm were manually prepared. Uniform sweet potato vines, each measuring 30 cm in length and containing 6 nodes, were carefully prepared. These vines were planted at a depth of 5 cm and at a spacing of 30 × 60 cm on April 5, 2022. NPSB rates were applied at the time of planting. The treatments were combined in a 3 × 4 factorial arrangement and laid out in a randomized complete block design (RCBD) with three replications. All other cultural practices were applied as recommended.

**Table 2:** Description of orange-fleshed sweet potato (OFSP) varieties used in the experiment.

Varieties	Year of release	Altitude adaptation (masl)	Days to Maturity	Skin color	Growth habit	Yield (t ha^−1^)	Dry matter content (%)	Unique characteristics	Utilization	Seed source
Alamura	2019	740–2200	120–150	Cream	Twining and spreading	25	31.80	Drought tolerance & disease resistance	Roasting, Baking, Mashing, frying & processing	HARC
Dilla	2019	740–2000	120–150	Cream	Twining and semi spreading	27.5	32.40	Drought tolerance & disease resistance	Roasting, Baking, Mashing, frying & processing	HARC
Kabode	2019	740–2000	90–120	Purple red	Non-twining and semi-erect	22.5	30.30	Drought tolerance & disease resistance	Roasting, Baking, Mashing, frying & processing	HARC

HARC: Hawassa Agricultural Research Center.

### 2.3 Sampling and data collection

Composite soil samples weighing around 1 kg were obtained at a depth of 0–30 cm from both study sites. Samples were air-dried, crushed, and sieved through a 2 mm sieve size and analyzed at the Areka Agricultural Research Center (AARC) soil laboratory. Selected soil physicochemical properties including soil pH, organic carbon, total nitrogen, available phosphorus, exchangeable potassium, sodium, calcium, and magnesium, available sulfur and boron, cation exchange capacity, soil texture, and bulk density. Detail methods used for soil analysis is indicated in Appendix Table 1.

Data on yield and yield-related parameters were collected at physiological maturity. Twelve plants were tagged from three interior rows of each plot, excluding the border rows. The root data taken includes root length (cm) (vertical length of the root measured from the tip to the scar of separation), root diameter (cm) (diameter of the root taken from the middle portion), average root weight (g root^−1^) (weight of individual roots), number of marketable roots per plant (the number of roots in a single plant), marketable root yield (t ha^−1^) (the weighed of the marketable category of roots), harvest index (%) (the ratio of total storage root yield to total biomass), root-to-shoot ratio (determined by dividing root biomass by corresponding aboveground biomass), root dry matter content (%) obtained from roots cut into smaller pieces and dried in a hot oven at 105 °C for 24 h) and total soluble solid (°Brix) (determined using a hand-held refractometer).

### 2.4 Data analysis

The data were tested for normality using Shapiro Wilk test before running an actual statistical analysis [15]. Then a principal component analysis (PCA) was performed to identify important parameters that contain most of the information. PCA was selected for its ability to condense multidimensional data into a more manageable set of variables, facilitating the detection of patterns and relationships within intricate datasets. By employing PCA, this study aims to unveil underlying structures and correlations among the myriad factors influencing sweet potato yield under varying NPSB rates. Justifying conclusions based on high-loading variables is essential as these variables hold significant explanatory power, offering crucial insights into the pivotal factors shaping the study's outcomes.

PCA transforms high-dimensional data into a lower-dimensional space by generating new uncorrelated variables (principal components) that capture maximum variance. It discerns critical features through the analysis of eigenvalues and eigenvectors, facilitating enhanced visualization and interpretation, particularly for complex datasets. Addressing multicollinearity, PCA constructs orthogonal components, diminishing data redundancy and refining subsequent analyses. The selection of principal components with the largest eigenvalues, explaining a substantial portion of the variance, simplifies the dataset for further analyses like predictive modeling and pattern recognition [16,17]. Accordingly, PCA reduced 14 parameters to nine. Data on root yield and yield components were recorded and analyzed using the GLM procedure in SAS 9.2 (SAS, 2004). The mean separation employed Bonferroni test at a 5 % probability level [18].

### 2.5 Agronomic efficiency

Agronomic efficiency (AE) measures the increase in yield per unit of nutrient applied and is indicative of the direct impact of the fertilizer on crop production and economic return. It was calculated using the following formula [19].

(1) $$\text{AE}=\frac{\;Y-Y_{0}}{F}*100$$

Where: AE = Agronomic Efficiency; Y = yield of the harvested portion of the crop with nutrient applied; Y_0_ = yield without nutrient applied; F = amount of nutrient applied. In this study, the harvested portion considered for the AE calculation was the yield of storage root.

### 2.6 Partial budget analysis

To consolidate the statistical analysis of the agronomic data, economic analysis was performed for each treatment. For economic evaluation, cost, return, and benefit-to-cost ratios were calculated according to the procedure given by Ref. [20]. Total variable cost (TVC) = sum of all variable costs in a given treatment. Storage root yield (SRY) = total yield harvested from 1 ha. Adjusted yield (AJY) = Tuber yield data was adjusted downward by 10 % to reflect the difference between experimental yield and farmers’ yield.

(2) Totalrevenue(TR)=AJY×unitpriceofthestorageroot

The gross field benefit for each treatment was calculated by multiplying the unit price by the adjusted yield.

(3) Netrevenue(NR)=totalrevenue(TR)−totalvariablecost(TVC)

The final line of the partial budget is the net benefits. It was calculated by subtracting the total costs that vary from the gross field benefits for each treatment.

(4) $$\text{Marginal}\;\text{rate}\;\text{of}\;\text{return}\;\left(\text{MRR}\right)=\frac{\Delta \text{NR}}{\Delta \text{TVC}}*100$$

Which is the marginal net benefit (i.e., the change in net benefit) divided by the marginal cost (that is, the change in cost).

## 3 Results and discussion

### 3.1 Soil physiochemical properties of the experimental sites

The soil analysis and the ratings of both sites are based on [14]. Arba Minch has lower nutrient levels than Areka, except for pH. According to EthioSIS [14], most nutrients are inadequate for crop productivity at both sites, highlighting the need for NPSB-containing fertilizer (Table 3).

**Table 3:** Pre-planting soil physiochemical properties of the experimental sites.

Physio-chemical properties	Sites			
	Areka		Arba Minch	
**Physical properties**	Values	textural class	Values	textural class
Clay (%)	34	Clay loam	30	Clay loam
Silt (%)	34		42	
Sand (%)	32		28	
BD (g/cm^3^)	1.23		1.16	
**Chemical properties**		**Status**		**Status**
(H_2_O 1:2.5)	5.16	Strongly acidic	6.78	Neutral
OM (%)	6.38	Optimum	4.05	Optimum
OC (%)	3.7	Medium	2.35	Medium
TN (%)	0.25	Low	0.18	Low
P_av_ (mg kg^−1^)	4.05	Very low	9.8	Very low
S_av_ (mg kg^−1^)	13.24	Low	10.72	Low
B_av_ (mg kg^−1^)	0.63	Low	0.54	Low
CEC (meq/100 g)	24.39	Optimum	19.7	Optimum
Ex. K (cmol kg^−1^)	2.35	Very low	1.75	Very low
Ex. Na (cmol kg^−1^	1.43	Optimum	1.06	Optimum
Ex. Ca (cmol kg^−1^)	5.07	Very low	4.53	Very low
Ex. Mg (cmol kg^−1^)	3.69	Very low	2.76	Very low

BD= Bulk Dunsity, OM= OrganicMatter, OC= Organic Carbon,TN = Total Nitrogen, P=Phosphorus, S = sulfur, B= Boron, CEC= Cation Exchange capacity, K = potassium, Na= Sodium, Ca= Calsium, Mg = Magnisium, Av = available, Ex = exchangeable.

### 3.2 Principal component analysis

**Fig. 2:**
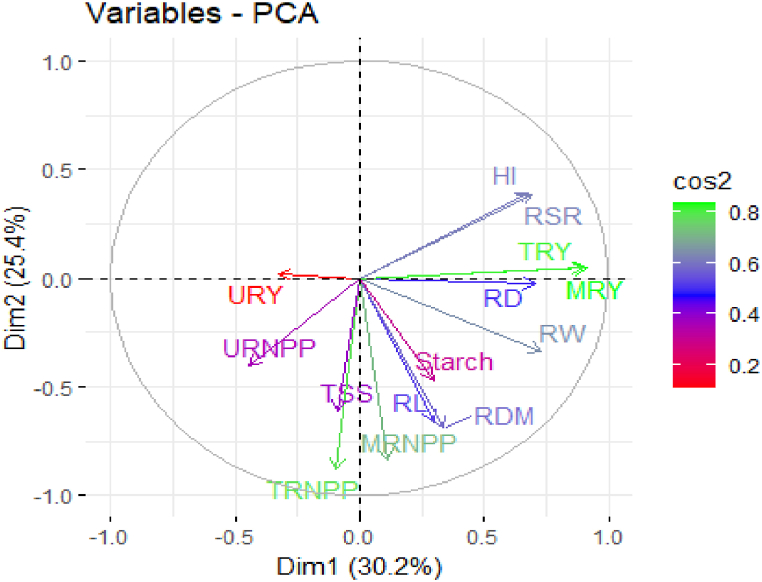
The loadings plot showing the distribution patterns of the measured characteristics over the first two principal components RL = Root Length (cm), RD = Root Diameter (cm), RW= Root Weight (g), MRNPP = Marketable Root Number Per Plant, URNPP= Unmarketable Root Number Per Plant, TRNPP = Total Root Number Per Plant, MRY = Marketable Root Yield (t ha^−1^), URY= Unmarketable Root Yield (t ha^−1^), TRY = Total Root Yield (t ha^−1^), HI= Harvest Index (%), RSR=Root to Shoot Ratio, RDM = Root Dry Matter (%), TSS = Total Soluble Solid (^0^Brix).

The Principal Component Analysis (PCA) loading plot (Fig. 2) shows the factor loadings of the first and second principal components. These components explain 25.4 % of the variation of the dataset. Narrow-angle features such as HI and RSR have a positive relationship, while right-angle features like HI and RW are unrelated. Wide-angle features, such as HI and URNPP, exhibit negative relationships. PCA loading plots help identify variable relationships and describe multivariate datasets [21].

**Fig. 3:**
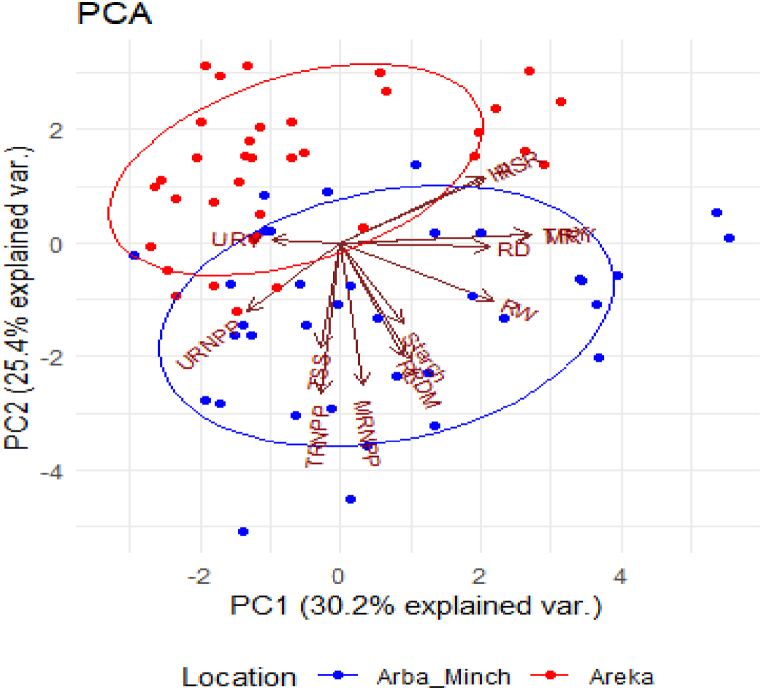
Biplot of the first two principal components showing the distribution patterns of factor loadings and scores of the measured characteristics at Arba Minch and Areka locations. Abbreviations as in Fig. 2. Blue and red dots represented row numbers for Arba Minch and Areka, respectively. (For interpretation of the references to color in this figure legend, the reader is referred to the Web version of this article.)

The biplot (Fig. 3) showes a clustering pattern of scores based on the two experimental sites, Arba Minch and Areka, for yield and yield component characters. Additionally, a smaller number of parameters were observed at the Areka site compared to Arba Minch. This variation could potentially be attributed to the characteristics of the varieties and/or the agroecology. OFSP varieties can have inherent genetic differences that influence their yield and yield components. These varietal characteristics may contribute to the observed variation in the PCA. For example, differences in root length, root diameter, root weight, and dry matter content agronomic, morphological and biochemical characteristics among the OFSP varieties (Kabode, Alamura, Dilla) could be a significant source of the variation captured by the principal components [21]. On the other hand, since the study was conducted at two different locations (Arba Minch and Areka), which have distinct agroecological conditions, such as soil type, climate, and other environmental factors, these agroecological differences between the two sites could also contribute to the observed variation in OFSP traits, as the plant's performance and response to the applied NPSB fertilizer may be influenced by the local environmental condition [22,23].

In this study, which involved 14 characters, three principal components (PCs) with eigenvalues greater than one (eigenvalues >1) were extracted (Table 4). The first six PCs, PC-1 to PC-3, account for 66.40 % of the total variation in the dataset (see Table 3). Among these, PC1 explains the highest variance at 30.21 %, followed by PC-2 at 25.43 %. The results of the PCA indicate that the first two PCs, PC-1 and PC-2, account for the majority of the variation in the data, specifically 55.63 %. While variables that show higher scores under these PCs can be useful in representing the treatment effects, it is important to note that not all variables will have high loadings on a single PC. Treatment effects might be spread across multiple PCs depending on the complexity of the data. This means that other factors besides the treatment effects, such as underlying biological relationships between the characters, interactions between characters, or inherent differences between the experimental units, can contribute to the variation captured by a PC [21]. Therefore, relying solely on high-loading variables from a single PC might not fully represent all treatment effects [17].

**Table 4:** Principal components and eigenvalues of the first three principal components (PCs) for fourteen characters of OFSP varieties at experimental sites Arba Minch and Areka.

Characters	PC-1	PC-2	PC-3
RL	0.299	**0.659**	−0.298
RD	**0.706**	0.026	−0.281
RW	**0.728**	0.339	−0.142
MRNPP	0.110	**0.837**	0.055
URNPP	−0.444	0.403	0.481
TRNPP	−0.093	**0.879**	0.247
MRY	**0.913**	−0.045	0.031
URY	−0.324	−0.018	**0.690**
TRY	**0.900**	−0.048	0.103
HI	**0.676**	−0.390	0.499
RSR	**0.690**	−0.382	0.489
RDM	0.336	**0.687**	0.039
TSS	−0.092	**0.610**	0.146
Starch content	0.293	0.469	0.167
Eigenvalue	4.229	3.559	1.513
Difference	0.669	2.046	0.588
Proportion	0.302	0.254	0.108
Cumulative	0.302	0.556	0.664

RL = Root Length (cm), RD = Root Diameter (cm), RW= Root Weight (g), MRNPP = Marketable Root Number Per Plant, URNPP= Unmarketable Root Number Per Plant, TRNPP = Total Root Number Per Plant, MRY = Marketable Root Yield (t ha^−1^), URY= Unmarketable Root Yield (t ha^−1^), TRY = Total Root Yield (t ha^−1^), HI= Harvest Index (%), RSR=Root to Shoot Ratio, RDM = Root Dry Matter (%), TSS = Total Soluble Solid (^0^Brix), In bold are characters with higher PC scores (>0.5 %).

Of the 14 characters examined, the majority (11) of them, namely RD, RW, MRY, TRY, HI, and RSR, show higher scores aligned with PC-1 (Table 4). However, five characters exhibit higher scores on PC-2. PCA analysis also indicates that TRNPP and TRY could be considered less important for further consideration. This is because TRNPP includes URNPP, and TRY contains URY, both of which make minor contributions to the variance explained in the PCA.

### 3.3 Effect of locations, NPSB rates, and varieties on yield-related characters of OFSP

#### 3.3.1 Root length, root diameter, and root weight

The three-way ANOVA revealed a significant interaction effect (p < 0.05) of site, variety, and NPSB rates on root length, diameter, and weight. Notably, at the Arba Minch site, the variety Alamura with 238.5 kg ha^−1^ of NPSB achieved the highest root length (27.23 cm). Similarly, variety Kabode at the same site produced a root diameter of 6.60 cm and a root weight of 500.58 g, both under the same NPSB treatment. In contrast, the Areka site showed lower performance, with variety Alamura yielding the shortest root length of 15.43 cm and variety Dilla the smallest diameter (3.41 cm) under unfertilized conditions. The lowest root weight (176.71 g) was recorded for variety Alamura treated with 79.5 kg ha^−1^ of NPSB at Areka (Table 5).

**Table 5:** Interaction effect of sites, varieties, and NPSB rates on mean values of root length, root diameter, and root weight of OFSP.

Sites	Varieties	NPSB (kg ha^−1^)	Root length (cm)	Root diameter (cm)	Root weight (g/root)
Arba Minch	Alamura	0.0	16.83^bc^	4.26^bc^	245.67^igh^
		79.5	18.70^bc^	4.79^bac^	341.97^cbd^
		159	24.93^ba^	4.71^bac^	358.20^cb^
		238.5	27.23^a^	4.78^bac^	324.93^ced^
	Kabode	0.0	17.03^bc^	3.73^bc^	288.67^fged^
		79.5	19.40^bac^	4.21^bc^	236.67^ighj^
		159	19.66^ehdfcg^	4.88^bac^	366.00^cb^
		238.5	21.16^bac^	6.60^a^	500.58^a^
	Dilla	0.0	20.86^bac^	3.58^bc^	259.07^fgh^
		79.5	23.86^ba^	4.65^bac^	302.96^fed^
		159	24.30^ba^	4.09^bc^	287.80^fged^
		238.5	22.63^bac^	4.81^bac^	320.64^ced^
Areka	Alamura	0.0	15.43^c^	3.94^bc^	186.70^kj^
		79.5	15.43^c^	3.78^bc^	176.71^k^
		159	20.36^bac^	3.67^bc^	197.80^ikj^
		238.5	19.86^bac^	4.23^ba^	237.40^ighj^
	Kabode	0.0	16.83^bc^	3.82^bc^	242.11^igh^
		79.5	18.06^bc^	5.29^bac^	192.00^ikj^
		159	18.16^bc^	5.70^ba^	384.10^b^
		238.5	17.40^bc^	4.71^bac^	281.56^fge^
	Dilla	0.0	15.63^c^	3.41^c^	215.00^ikhj^
		79.5	15.53^c^	3.68^bc^	184.39^kj^
		159	17.76^bc^	3.69^bc^	226.47^ikhj^
		238.5	22.76^bac^	4.74^bac^	235.70^ighj^
Mean			19.57	4.41	274.71
CV (%)			12.56	15.19	5.93
Bonferroni (0.05)			8.16	2.18	54.93

CV: Coefficient of Variation. Means followed by the same letter in a column are not significantly different at a 5 % probability level.

The differences in root length, diameter, and weight can be attributed to variations in environmental conditions, genetics, management practices, and nutrient availability. These factors interact and contribute to the final size and weight of sweet potato roots. Moreover, Arba Minch site had favorable soil conditions, including lower bulk density and better aeration, promoting sweet potato root growth. Genetic inheritance among varieties may also contribute to the observed variations. In Etana and Alo [24,25] reports, significant differences in root length among varieties were observed, consistent with the current study.

Our findings agree with previous research by Refs. [6,24], they all reported significant variations in root diameter and weight due to interactions between varieties and NPSB fertilizer. These studies demonstrate the influence of different varieties on the average root diameter and weight of sweet potatoes.

#### 3.3.2 Marketable root number per plant and marketable root yield

The three-way interaction of site, variety, and fertilizer significantly influenced the marketable root number per plant and marketable root yield (p < 0.05). At the Arba Minch site, varieties Alamura and Kabode, treated with 238.5 kg ha⁻^1^ of NPSB fertilizer, achieved the highest marketable root number per plant (9.17) and a marketable root yield (49.84 t ha⁻^1^. In contrast, unfertilized plots for the varieties Kabode and Alamura produced the lowest marketable root number per plant (2.30) and yield (16.79 t ha^−1^) at the Areka and Arba Minch experimental sites, respectively (Table 6).

**Table 6:** Interaction effect of site, varieties, and NPSB rates on mean values of marketable root number per plant and marketable root yield of OFSP.

Sites	Varieties	NPSB (kg ha^−1^)	Marketable root number per plant	Marketable root yield
Arba Minch	Alamura	0	5^ebdac^	16.79^f^
		79.5	5.93^ebdac^	29.47^ebdacf^
		159	7.70^bac^	40.23^ebdac^
		238.5	9.17^a^	24.80^edcf^
	Kabode	0	6.37^bdac^	18.64^f^
		79.5	4.80^ebdac^	29.39^ebdacf^
		159	5.87^ebdac^	46.42^ba^
		238.5	5.57^ebdac^	49.84^a^
	Dilla	0	6.80^bdac^	19.79^ef^
		79.5	6.86^bdac^	27.30^ebdcf^
		159	7.70^bdac^	45.03^bac^
		238.5	5.43^ebdac^	44.86^bac^
Areka	Alamura	0	3.90^edc^	23.47^edf^
		79.5	4.76^ebdac^	22.44^edf^
		159	8.56^ba^	24.76^edcf^
		238.5	5.00^ebdac^	24.84^edcf^
	Kabode	0	2.30^e^	18.52^f^
		79.5	3.00^ed^	41.47^bdac^
		159	4.53^ebdc^	47.25^ba^
		238.5	5.26^ebdac^	44.90^bac^
	Dilla	0	4.96^ebdac^	19.58^ef^
		79.5	4.16^ebdc^	31.75^ebdacf^
		159	3.06^ed^	26.96^ebdcf^
		238.5	4.10^edc^	23.07^edf^
Mean			5.45	30.90
CV (%)			24.89	21.16
Bonferroni (0.05)			4.41	21.34

CV: Coefficient of Variation. Means followed by the same letter in a column are not significantly different at a 5 % probability level.

The higher marketable root number per plant and marketable root yield of sweet potatoes in Arba Minch compared to the Areka experimental site can be attributed to factors such as warmer temperatures, longer day lengths, consistent rainfall, fertile soils, and lower pest and disease pressure. These favorable environmental conditions in tropical regions promote better root development, enhanced photosynthesis, and improved nutrient uptake, resulting in increased marketable root production and overall yield. Study by Etana [24] showed that applying fertilizers increased marketable root number per plant. The results of the current study were comparable to those of Gobena et al. [26] findings for Beletech and Awassa-83 varieties, but significantly higher overall. These findings confirm the impact of variety and fertilizer on marketable root yield, as demonstrated by Refs. [24,27,28]. The studies highlight the variation in yield between sweet potato varieties and the positive effect of fertilizer application, particularly phosphorus. The results of the current study exceeded reported the report yields for Kulfo and Hawassa-83 varieties, indicating potential for improved yields.

#### 3.3.3 Harvest index and root-to-shoot ratio

**Fig. 4:**
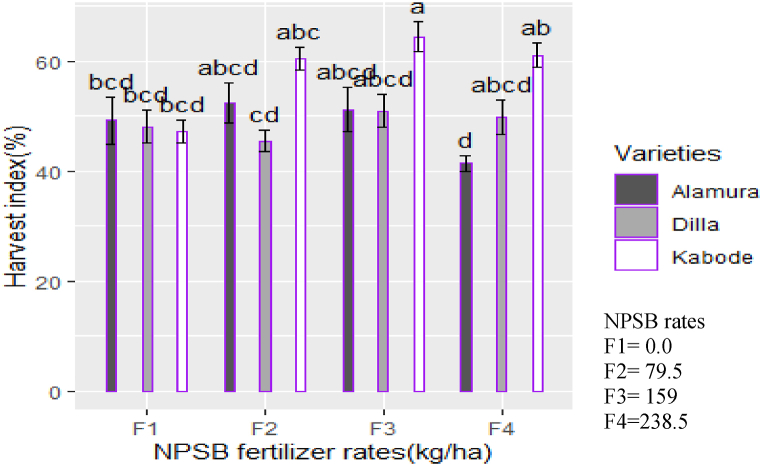
Interaction effect of NPSB rates and varieties on harvest index of OFSP.

**Fig. 5:**
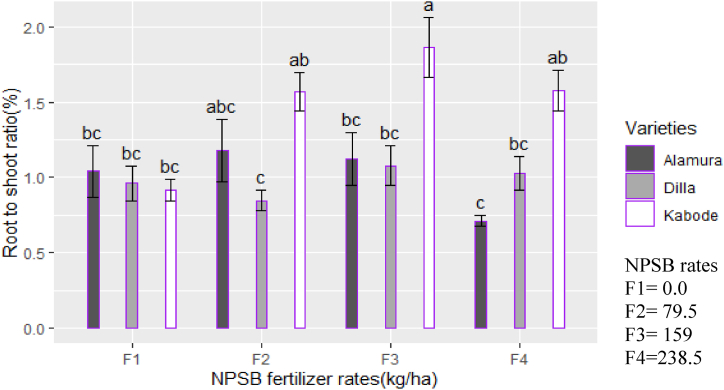
Interaction effect of NPSB rates and varieties on root-to-shoot ratio of OFSP.

The interaction of variety and NPSB rates significantly affected the harvest index and root-to-shoot ratio (p < 0.05). Variety Kabode, treated with 159 kg ha⁻^1^ of NPSB, achieved the highest values for both the harvest index (Fig. 4) and root-to-shoot ratio (Fig. 5). Conversely, variety Alamura, treated with 238.5 kg ha⁻^1^ of NPSB, recorded the lowest mean values for these metrics. The difference might be attributed to differences in the genetic traits of sweet potato varieties affect resource allocation between aboveground and belowground plant parts. Under different fertilizer treatments, these varieties show varied responses in biomass allocation. Specific fertilizers can improve root growth in certain varieties, leading to a higher harvest index and increased biomass in desired storage roots [28]. Variety-fertilizer interactions also impact the root-to-shoot ratio and some varieties naturally tend towards larger root systems, which can be further improved with specific fertilizer treatments [29].

On the other hand, Higher NPSB application can influence the microbial community in the rhizosphere of Kabode plants, potentially enhancing nutrient solubilization and mineralization processes. This shift in the rhizosphere microbiome could lead to improved nutrient availability for Kabode plants compared to Alamura. Furthermore, NPSB application may stimulate the expression of genes associated with nutrient uptake and transport in Kabode, optimizing the plant's ability to acquire and distribute nutrients efficiently. These combined effects highlight the intricate interplay between NPSB application, plant-microbe interactions, and gene regulation, ultimately shaping nutrient uptake and partitioning dynamics in Kabode relative to Alamura [28,29].

Different studies have shown that the interaction between sweet potato varieties and fertilizer rates significantly affects the harvest index. Etana, and Mekonnen et al. [17,22] reported a significant interaction, while Darko et al. [30] found no influence. Excessive nutrient levels promote shoot growth but hinder dry matter transport to the roots, reducing the root-to-shoot ratio and final yield [31]. In vine production, higher fertilizer rates can increase shoot yield at the expense of storage root yield [32,33].

Root dry matter and total soluble solid.

The three-way interaction of site, variety, and NPSB rates significantly influenced root dry matter (p < 0.05) and total soluble solids (TSS) (p < 0.01). At the Arba Minch site, variety Dilla, treated with 159 kg ha⁻^1^ of NPSB, exhibited the highest root dry matter at 95.10 %, while variety Alamura, treated with 238.5 kg ha⁻^1^ of NPSB, recorded the highest TSS at 17.66 °Brix. On the other hand, the control plots at the Areka site showed the lowest values, with variety Alamura having a root dry matter of 22.69 % and variety Kabode recording a TSS of 10.50 °Brix. Overall, all three varieties at Areka displayed statistically similar but lower root dry matter values compared to those at Arba Minch (Table 7).

**Table 7:** Interaction effect of site, variety, and fertilizer rate on root dry matter and total soluble solid of OFSP.

Sites	Varieties	NPSB (kg ha^−1^)	Root dry matter (%)	TSS (^0^Brix)	
Arba Minch	Alamura	0.0	77.17^bc^	15.00^bdac^	
		79.5	79.02^bac^	12.00^bdc^	
		159	80.42^bac^	15.33^bac^	
		238.5	80.18^bac^	17.66^a^	
	Kabode	0.0	83.21^bac^	14.33^bdac^	
		79.5	77.62^bc^	15.00^bdac^	
		159	87.41^ba^	12.00^bdc^	
		238.5	75.52^bc^	15.00 ^bdac^	
	Dilla	0.0	78.08^bc^	15.00 ^bdac^	
		79.5	69.23^c^	15.00 ^bdac^	
		159	95.10^a^	15.00 ^bdac^	
		238.5	81.81^bac^	13.33^bdac^	
Areka	Alamura	0.0	22.69^d^	13.33^bdac^	
		79.5	35.52^d^	16.33^ba^	
		159	31.48^d^	11.00^dc^	
		238.5	28.26^d^	12.50^bdc^	
	Kabode	0.0	31.75^d^	10.50^d^	
		79.5	25.09^d^	12.00^bdc^	
		159	27.37^d^	11.50^dc^	
		238.5	29.13^d^	11.66^bdc^	
	Dilla	0.0	29.07^d^	14.33^bdec^	
		79.5	28.91^d^	14.00^bdac^	
		159	26.53^d^	12.00^bdc^	
		238.5	28.21^d^	11.66^bdc^	
Mean			54.53	13.56	
CV (%)			9.42	10.36	
Bonferroni (0.05)			16.95	4.68	

CV: Coefficient of Variation. Means followed by the same letter in a column are not significantly different at a 5 % probability level.

The decrease in root dry matter with increasing NPSB rates at the Arba Minch site may be attributed to a high level of NPSB fertilizer, which could reduce the dry matter of sweet potato root due to imbalances and disruptions in nutrient uptake and allocation [29]. Excess nitrogen and phosphorus from the fertilizer can stimulate excessive shoot growth, diverting resources away from root development and dry matter accumulation in the storage roots [22]. Additionally, imbalances in sulfur and boron, which are essential micronutrients for sweet potato growth, can negatively affect root function and nutrient uptake, further affecting the accumulation of dry matter in the storage roots [34,35]. As a result, high levels of NPSB fertilizer can hinder the overall growth and productivity of sweet potato storage roots. High levels of NPSB fertilizer levels could also reduce total soluble solids of sweet potato by promoting excessive vegetative growth and inhibiting carbohydrate accumulation in storage roots. Imbalances in phosphorus, sulfur, and boron further disrupt sugar synthesis and accumulation, resulting in decreased concentrations of total soluble solids [[31], [32], [33],^36^].

This study is comparable with the findings of Etana [24], showing that the interaction between variety and fertilizer affects the dry matter content sweet potato root. The impact of genotype and environment on root dry matter content was also significant [37,38]. However, NafiHa et al. [39] reported no influence of fertilizer on TSS values, contrasting with the current study. Dias and Dresch [40] observed TSS values ranging from 7.9 to 9.2 ^0^Brix and concluded that organic fertilization did not affect sweet potato root TSS. The contrasting results with the previous study could be attributed to various factors such as differences in soil fertility, climate conditions, crop varieties, application methods, and measurement techniques. Potential limitations in prior studies might include inadequate control of confounding variables, small sample sizes, or variations in experimental designs. Our study aims to address these discrepancies by considering a broader range of agro-ecological zones in southern Ethiopia, offering a more comprehensive understanding of how NPSB fertilizer rates affect TSS values in OFSP sweet potato cultivation.

#### 3.3.4 Agronomic Efficiency

The agronomic efficiency (AE) was significantly influenced by the interaction of site, variety, and fertilizer rates (Table 8). At the Areka site, variety Kabode achieved the highest AE of 288.69 with 79.5 kg ha⁻^1^ of NPSB, followed by the same variety with 159 kg ha⁻^1^, which had an AE of 180.72. Conversely, variety Alamura recorded the lowest AE of −12.90 when treated with 79.5 kg ha⁻^1^ of NPSB at Areka. At the Arba Minch site, the highest AE of 174.70 was again noted for variety Kabode treated with 159 kg ha⁻^1^ of NPSB, while variety Alamura, with 238.5 kg ha⁻^1^ of NPSB, exhibited the lowest AE at 33.59.

**Table 8:** Agronomic efficiency for mean values of treatment interaction of OFSP varieties and NPSB rates for Arba Minch and Areka experimental sites.

Treatments		NPSB rates (kg ha^−1^)		
Sites	Varieties	79.5	159	238.5
Areka	Alamura	−12.90^c^	8.11^bc^	5.77^bc^
	Kabode	288.69^a^	180.72^ab^	110.59^abc^
	Dilla	153.08^abc^	46.41^bc^	14.64^bc^
Arba Minch	Alamura	159.58^abc^	147.48^abc^	33.59^bc^
	Kabode	135.14^abc^	174.70^ab^	130.82^abc^
	Dilla	94.49^bc^	158.71^abc^	105.09^bc^
Mean	107.48			
CV (%)	53.57			
Bonferroni (0.05)	181.94			

CV: Coefficient of Variation. Means followed by the same letter in a column are not significantly different at a 5 % probability level.

The agronomic efficiency of sweet potato is reduced with increasing levels of NPSB fertilizer due to factors such as excessive nitrogen that promote vegetative growth at the expense of root development, nutrient imbalances that disrupt nutrient uptake and utilization, and potential losses of nutrient through leaching or runoff [41]. These factors contribute to the decreased in the efficiency converting applied nutrients into harvestable yields. Optimizing fertilizer application rates is crucial to strike a balance between plant growth and nutrient utilization, thus maximizing agronomic efficiency in sweet potato production [42,43,44].

#### 3.3.5 Partial budget analysis

The partial budget analysis indicated that variety Kabode, with an application of 238.5 kg ha⁻^1^ of NPSB, yielded the highest net benefit of 342,856.6 ETB ha⁻^1^ at the Arba Minch site, demonstrating an acceptable marginal rate of return (Table 9). Following closely, the same variety treated with 159 kg ha⁻^1^ of NPSB generated net benefits of 328,138.4 ETB ha⁻^1^ at Areka and 322,162.4 ETB ha⁻^1^ at Arba Minch, both with acceptable marginal rates of return. In contrast, the lowest net benefit of 116,686 ETB ha⁻^1^ was recorded for variety Alamura without NPSB treatment at Arba Minch. These findings highlight that while agronomic efficiency varied, the highest net benefits consistently arose from different NPSB rates applied to variety Kabode. This implies that AE do not necessarily translate into productivity or financial outcomes as AE increases with the reduction of fertilizer inputs as in the formula Fixen et al. and Govindasamy [14,45]. Therefore, our findings imply that growers need to consider agronomic efficiencies and financial returns from investment in selecting productive varieties and determining fertilizer inputs.

**Table 9:** Partial budget analysis for mean values of treatment interactions of OFSP varieties and NPSB rates for Arba Minch and Areka experimental sites.

Sites	Varieties	NPSB (kg ha^−1^)	Unadju Yield (t ha^−1^)	Adju yield (t ha^−1^) by 10 %	TVC (ETB ha^−1^)	Gross benefit (ETB ha^−1^)	Net benefit (ETB ha^−1)^	MRR (%)
Arba Minch	Alamura	0	16.79	15.11	4202	120888	116686	
		79.5	29.47	26.52	8131.8	212184	204052.2	2223.17
		159	40.23	36.20	12061.6	289656	277594.4	1871.39
		238.5	24.80	22.32	15991.4	178560	162568.6	D
	Kabode	0	18.64	16.77	4202	134208	130006	
		79.5	29.39	26.45	8131.8	211608	203476.2	1868.56
		159	46.42	41.77	12061.6	334224	322162.4	3020.15
		238.5	49.84	44.85	15991.4	358848	342856.6	526.59
	Dilla	0	19.79	17.81	4202	142488	138286	
		79.5	27.30	24.57	8131.8	196560	188428.2	1275.94
		159	45.03	40.52	12061.6	324216	312154.4	3148.41
		238.5	44.86	40.37	15991.4	322992	307000.6	D
Areka	Alamura	0	23.47	21.12	4202	168984	144782	
		79.5	22.44	20.19	8131.8	161568	153436.2	D
		159	24.76	22.28	12061.6	178272	166210.4	325.05
		238.5	24.84	22.35	15991.4	178848	162856.6	D
	Kabode	0	18.52	16.66	4202	133344	129142	
		79.5	41.47	37.32	8131.8	298584	290452.2	4104.79
		159	47.25	42.52	12061.6	340200	328138.4	958.98
		238.5	44.90	40.41	15991.4	323280	307288.6	D
	Dilla	0	19.58	17.62	4202	140976	136774	
		79.5	31.75	28.57	8131.8	228600	220468.2	2129.73
		159	26.96	24.26	12061.6	194112	182050.4	D
		238.5	23.07	20.76	15991.4	166104	150112.6	D

Unadju = Unadjusted yield; Adju = Adjested yield; TVC = Total Variable Cost; MRR = Marginal Rate of Return, D = Dominant.

The net benefit of sweet potato production is influenced by the yield obtained, which is impacted by various factors including environmental conditions, varieties differences, and fertilizer levels. High yield contributes to a higher net benefit, while low yield leads to a reduced net benefit [43,46,47].

## 4 Conclusions

Orange-fleshed sweet potato is a valuable source of cost-effective vitamin A in the form of β-carotenes. However, poor agronomic practices, insufficient fertilizer information, and a lack of high-yielding, nutrient-rich varieties pose major challenges in farmers' fields. Therefore, optimizing the application of blended fertilizer is crucial for improving production, productivity, and nutritional value of orange-fleshed sweet potato varieties in areas with vitamin A deficiencies. This study revealed that NPSB rates, locations, varieties, and their two and three-way interactions had a great impact on most of the yield, and yield components. Means of root length, root diameter, root weight, marketable root yield, and root dry matter content were highly significant (p < 0.01) in the three-way interaction between sites, OFSP varieties, and NPSB rates. At Arba Minch site, increasing NPSB rates led to significant increases in marketable root yield and root weight, with root dry matter peaking at 159 kg ha^−1^ NPSB. Similarly, at Areka site, marketable root yield and root weight increased up to 159 kg ha^−1^ NPSB. The Kabode variety, when treated with 238.5 kg ha^−1^ NPSB at Arba Minch and 159 kg ha^−1^ NPSB at Areka, achieved the highest marketable root yields. In terms of agronomic efficiency, At the Areka experimental site, variety Kabode showed agronomic efficiencies of 288.67 kg/kg and 180.69 kg/kg at NPSB rates of 79.5 kg ha^−1^ and 159 kg ha^−1^ respectively. At the Arba Minch site, the highest agronomic efficiency (174.71 kg/kg) was achieved by the Kabode variety treated with 159 kg ha^−1^ NPSB. The net benefit analysis also indicated that Kabode with 238.5 kg ha^−1^ NPSB resulted in the highest net benefit (342,856.6 ETB/ha) and acceptable marginal rate of return at Arba Minch site. Similarly, using 159 kg ha^−1^ NPSB, Kabode variety yielded net benefits of 328,138.4 ETB/ha and 322,162.4 ETB/ha with acceptable marginal rates of return at Areka and Arba Minch sites respectively. Generally, Variety Kabode outperformed Alamura and Dilla in both sites. Kabode treated with 79.5 kg ha^−1^ NPSB is recommended for Areka, while 159 kg ha^−1^ NPSB is recommended for Arba Minch and other similar areas. While these findings provide valuable understanding, the authors caution that further research is needed across multiple seasons and locations, including additional OFSP varieties, to obtain more reliable and generalizable information. This is necessary to ensure the recommended NPSB rates and OFSP varieties can be confidently applied to vastly different environments without the need for additional trials.

## CRediT authorship contribution statement

**Amelewerk Gizachew:** Writing – review & editing, Writing – original draft, Visualization, Methodology, Investigation, Conceptualization. **Sabura Shara:** Writing – review & editing, Methodology, Conceptualization. **Asfaw Kifle:** Writing – review & editing, Conceptualization.

## Ethical statements

Not applicable for this research as it does not involve animal or human subjects for data collection.

## Data availability statement

Data will be available on request from the corresponding author.

## Funding statement

Financial support for this work was granted by the 10.13039/501100003081Ethiopian Ministry of Education through Arba Minch University.

## Declaration of competing interest

The authors declare that they have no known competing financial interests or personal relationships that could have appeared to influence the work reported in this paper.
