# Supplementary material for: Impact of NPSB fertilizer on yield of orange-fleshed sweet potato (Ipomoea batatas (L.) Lam.) varieties in Southern Ethiopia's agro-ecological zones
Source: Heliyon. 2024 Nov 23;10(23):e40660. doi: 10.1016/j.heliyon.2024.e40660 (PMC11626010; doi:10.1016/j.heliyon.2024.e40660)
Supplement: Multimedia component 1 [file mmc1.docx]

Appendix Table 1. Methods used for soil analysis

| Physio-chemical properties | Chemical used for nutrients extraction | Methods | References |
| --- | --- | --- | --- |
| **Physical properties** |  |  |  |
| Soil texture | Sodium hexametaphosphate | Hydrometer method | [48] |
| Bulk density | Core sampler | Oven dry | [49] |
| **Chemical properties** | | | |
| pH (H_2_O 1:2.5) | 1:2.5 water suspension | Glass Electrode Method | [50] |
| OC (%) | Potassium dichromate-sulfuric acid | Walkley-Black method | [51] |
| OM (%) |  | Calculated by multiplying the percentage of organic carbon (OC) by the factor of 1.724 | [52] |
| TN (%) | Sulfuric acid and a catalyst (like selenium) | Kjeldahl method or Dumas combustion method | [53] |
| P_av_ (mg kg^-1^) | Sodium bicarbonate | Olsen extraction method | [54] |
| S_av_ (mg kg^-1^ | Calcium chloride | Turbidimetric methods |  |
| B_av_ (mg kg^-1^) | Dilute HCl | Dilute HCl extraction method |  |
| CEC (meq/100 g) | Ammonium acetate | Distillation followed by titration | [55] |
| Ex. K (cmol kg^-1^) and Ex. Na (cmol kg^-1^ | Ammonium acetate | Flame photometer | [56] |
| Ex. Ca (cmol kg^-1^) and Ex. Mg (cmol kg^-1^) | Ammonium acetate | Atomic absorption spectroscopy | [57] |

BD= Bulk Dunsity, OM= OrganicMatter, OC= Organic Carbon,TN= Total Nitrogen, P=Phosphorus, S= sulfur, B= Boron, CEC= Cation Exchange capacity, K= potassium, Na= Sodium, Ca= Calsium, Mg= Magnisium, Av = available, Ex = exchangeable
